# Survival factors of hospitalized out-of-hospital cardiac arrest patients in Taiwan: A retrospective study

**DOI:** 10.1371/journal.pone.0191954

**Published:** 2018-02-08

**Authors:** Chung-Yu Lai, Fu-Huang Lin, Hsin Chu, Chih-Hung Ku, Shih-Hung Tsai, Chi-Hsiang Chung, Wu-Chien Chien, Chun-Hsien Wu, Chi-Ming Chu, Chi-Wen Chang

**Affiliations:** 1 Graduate Institute of Medical Sciences, National Defense Medical Center, Taipei City, Taiwan; 2 School of Public Health, National Defense Medical Center, Taipei City, Taiwan; 3 Graduate Institute of Aerospace and Undersea Medicine, National Defense Medical Center, Taipei City, Taiwan; 4 Department of Health Industry Management, Kainan University, Taoyuan City, Taiwan; 5 Department of Emergency Medicine, Tri-Service General Hospital, National Defense Medical Center, Taipei City, Taiwan; 6 Division of Cardiology, Tri-Service General Hospital, National Defense Medical Center, Taipei City, Taiwan; 7 Department of Public Health, China Medical University, Taichung City, Taiwan; 8 Big Data Research Center, Fu-Jen Catholic University, New Taipei City, Taiwan; 9 School of Nursing, College of Medicine, Chang Gung University, Taoyuan City, Taiwan; 10 Department of Pediatrics, Chang Gung Memorial Hospital, Taoyuan City, Taiwan; Yokohama City University, JAPAN

## Abstract

The chain of survival has been shown to improve the chances of survival for victims of cardiac arrest. Post-cardiac arrest care has been demonstrated to significantly impact the survival of out-of-hospital cardiac arrest (OHCA). How post-cardiac arrest care influences the survival of OHCA patients has been a main concern in recent years. The objective of this study was to assess the survival outcome of hospitalized OHCA patients and determine the factors associated with improved survival in terms of survival to discharge. We conducted a retrospective observational study by analyzing records from the National Health Insurance Research Database of Taiwan from 2007 to 2013. We collected cases with an *International Classification of Disease Clinical Modification*, *9*^*th*^ *revision* primary diagnosis codes of 427.41 (ventricular fibrillation, VF) or 427.5 (cardiac arrest) and excluded patients less than 18 years old, as well as cases with an unknown outcome or a combination of traumatic comorbidities. We then calculated the proportion of survival to discharge among hospitalized OHCA patients. Factors associated with the dependent variable were examined by logistic regression. Statistical analysis was conducted using SPSS 22 (IBM, Armonk, NY). Of the 11,000 cases, 2,499 patients (22.7%) survived to hospital discharge. The mean age of subjects who survived to hospital discharge and those who did not was 66.7±16.7 and 71.7±15.2 years, respectively. After adjusting for covariates, neurological failure, cardiac comorbidities, hospital level, intensive care unit beds, transfer to another hospital, and length of hospital stay were independent predictors of improved survival. Cardiac rhythm on admission was a strong factor associated with survival to discharge (VF vs. non-VF: adjusted odds ratio: 3.51; 95% confidence interval: 3.06–4.01). In conclusion, cardiac comorbidities, hospital volume, cardiac rhythm on admission, transfer to another hospital and length of hospital stay had a significant positive association with survival to discharge in hospitalized OHCA patients in Taiwan.

## Introduction

Cardiac arrest is defined as the ‘sudden cessation of heart beat and the abrupt loss of cardiac mechanical activity in a person who may or may not have been diagnosed with heart disease, confirmed by the absence of a detectable pulse and unresponsiveness of apnea’ [1–3]. Depending on the scene, cardiac arrest can be categorized as either out-of-hospital cardiac arrest (OHCA) or in-hospital cardiac arrest (IHCA) [4, 5]. There are approximately 350,000 and 275,000 adult OHCA patients annually in the United States and Europe, respectively [6–8]. OHCA remains a public health concern.

The chain of survival provides a standard protocol for resuscitation and treatment. A strong chain of survival has been shown to improve the chances of survival and recovery for victims. In 2010, the chain of survival for adult OHCA was extended to five links: early recognition, early cardiopulmonary resuscitation (CPR), rapid defibrillation, emergency medical services (EMS), advanced life support and in-hospital post-cardiac arrest care. Rapid transport to the hospital for advanced care is determined by the classification of diseases and clinical characteristics [9], and post-arrest care has been demonstrated to significantly impact the survival of OHCA patients.

Approximately 60% of OHCA events are treated by EMS [10, 11]. The pooled rate of survival to admission was 23.4% [12]. The reported rate of survival to hospital discharge ranges from 0.2% to 37.8% [13–16]; however, a meta-analysis yielded a pooled rate of 7.6% [12]. Even when EMS was activated promptly, the rate of survival to discharge among EMS-treated OHCA patients was approximately 10% [17].

Studies have documented a variety of factors associated with prognosis after OHCA, including patient variables (e.g., age, gender, and comorbidities) [18, 19], event variables (e.g., initial cardiac rhythm, time to CPR and defibrillation) [7, 18] and hospital variables (e.g., level and post-cardiac arrest care) [20].

The concept of how post-cardiac arrest care influences the survival of OHCA patients admitted to a hospital has been highlighted in recent years. Yet, to the best of our knowledge, only a few hospital-based studies have investigated this issue in Taiwan. To bridge this gap, we analyzed the health insurance database to examine the survival outcome of hospitalized OHCA patients. Next, we identified factors associated with improved survival in terms of survival to discharge.

## Materials and methods

### Design and resources

This was a long-term, retrospective, nationwide population-based study in Taiwan. The National Health Insurance (NHI) Program is a publicly funded, single-tier, universal health insurance system that has been in operation since 1995 and is available to all citizens of Taiwan. The NHI involves 97% of medical providers and provides health services to 99% of the population [21]. The NHI Research Database (NHIRD) was the source of data in this study and medical diagnoses recorded according to the *International Classification of Disease Clinical Modification*, *9*^*th*^ *revision codes* (ICD-9-CM) [22].

After the de-identification and encryption process, the National Health Research Institute (NHRI) recompiled medical claims and provided NHIRD data to the researchers after careful protocol evaluation. Each patient was assigned a non-specific code and single hospital identifier unique within the database; this information cannot be used to trace individual subjects or hospitals outside the database to protect privacy.

We examined three different datasets: inpatient expenditures by admissions (DD), registry for contracted medical facilities (HOSB) and registry for contracted beds (BED) starting on January 1, 2007 and ending on December 31, 2013. The comprehensive NHIRD is regularly maintained and cross-checked to confirm the validity and accuracy of diagnoses [21, 22].

### Inclusion criteria

The DD dataset only contained information of hospitalized patients and did not contain the medical details of those in the emergency department (ED). Each case had one primary diagnostic code, four secondary diagnostic codes, and five procedure codes. We collected cases with a primary diagnosis of ‘ventricular fibrillation (VF)’ or ‘cardiac arrest’ using the ICD-9-CM code 427.41 or 427.5. We specifically defined cases with a primary diagnostic code of ICD-9-CM 427.41 as VF cardiac rhythm [23–27]. If patients experienced multiple cardiac arrest events during hospitalization, only the primary code of the first event was retrieved; for the purpose of this study, these patients were defined as hospitalized OHCA patients who only survived beyond ED resuscitation and were stable enough for formal admission, not including patients who died in the ED.

In total, 12,870 cases were selected from the NHIRD; 1,870 cases were excluded due to one of following selection criteria: age < 18 years old (n = 205), unknown survival outcome or continuous hospitalization in the study period (n = 497), or combination with traumatic comorbidities (ICD-9-CM codes: 800 to 999, injury external codes: E800 to E999) (n = 1328). Ultimately, 11,000 hospitalized OHCA patients were entered into the analysis.

### Primary outcome and operational definition

The primary outcome was the proportion of survival to discharge among OHCA patients admitted to a hospital. Details in three major categories of variables were examined: patients’ demographic and clinical data, hospital data, and event data.

Among the demographic and clinical characteristics, the variables included age, gender, and comorbidities. There were covariates in the hospital data, including the hospital level (medical center, regional, and local), geographic area (northern, central, southern, eastern, and offshore regions of Taiwan and Taipei city), teaching hospital status (yes/no), hospital size [quartiles of beds and intensive care unit (ICU) beds]. Event data included cardiac rhythm on admission (VF vs. non-VF), transfer to another hospital (yes vs. no), length of hospital stay and calendar year (2007 to 2010 vs. 2011 to 2013).

Severe comorbidities probably affecting survival were also defined by ICD-9-CM codes from four secondary diagnostic codes and five procedure codes. We focused on the following comorbidities: liver failure (ICD-9-CM codes 570, 572.2, and 573.3), heart failure (ICD-9-CM codes 428, 458.0, 458.8, 458.9, 785.5, 785.51, 785.59, and 796.3), renal failure (ICD-9-CM codes 39.95, 580, 584, and 585), respiratory failure (ICD-9-CM codes 96.7, 518.81, 518.82, 518.85, 786.09, and 799.1), neurological failure (ICD-9-CM codes 89.14, 293, 348.1, 348.3, 780.01, and 780.09), metabolic problem (ICD-9-CM code 276.2), diabetes mellitus (ICD-9-CM code 250), hypertension (ICD-9-CM codes 401 to 405), stroke (ICD-9-CM codes 430 to 438), coronary artery disease (CAD) (ICD-9-CM codes 410 to 414), valvular heart disease (ICD-9-CM codes 393 to 398, 424), cardiomyopathy (ICD-9-CM code 425), atrial flutter/fibrillation (ICD-9-CM code 427.3), pulmonary embolism (ICD-9-CM code 415.1), chronic obstructive pulmonary disease (COPD) (ICD-9-CM codes 490 to 496), asthma (ICD-9-CM code 493), and malignancy (ICD-9-CM codes 140 to 239) [23, 28].

### Statistical analysis

We report the data of survival to hospital discharge among hospitalized OHCA patients. Descriptive statistics (the mean ± standard deviation and proportion) are presented to describe continuous and categorical variables. We identified differences between survivors and non-survivors to discharge in demographic, clinical, hospital, and event characteristics using univariate tests. Continuous data were analyzed by independent *t* tests. Categorical and binary variables were analyzed by *chi-square* tests. Variables with two-tailed *P* values <0.05 determined by univariate tests were included in a multivariate logistic regression model.

Multivariate logistic regression was undertaken to determine the contributors to the dependent variable. All covariates with a *P* value <0.05 in the main effects model were considered significant by a backward stepwise selection method. All data were managed using SPSS version 22 (IBM, Armonk, NY).

### Ethical approval

This study was approved by the ethical committee of the Institutional Review Board of Tri-Service General Hospital in Taipei City, Taiwan (TSGHIRB No. 1-105-05-136).

## Results

A total of 11,000 cases of hospitalized OHCA from 2007 to 2013 were collected. In all, 2,499 patients (22.7%) survived to hospital discharge. As shown in Table 1, the mean age of subjects who survived to hospital discharge and those who did not was 66.7±16.7 and 71.7±15.2 years, respectively (*P*<0.001). The percentage of males was 59.0% in the discharge group and 58.2% in the non-discharge group. Respiratory failure was the most common problem among the clinical characteristics. Heart, renal, respiratory, and metabolic failure were less frequent in the discharge group; inversely, neurological failure was more likely in the discharge group (discharge: 24.5%; non-discharge: 17.2%; *P*<0.001). There were higher proportions of hypertension, CAD, valvular heart disease, cardiomyopathy and atrial flutter/fibrillation in patients who survived to discharge. However, malignancy was less frequent in the discharge group (discharge: 4.6%; non-discharge: 8.1%; *P* = 0.001).

**Table 1:** Demographic and clinical characteristics of hospitalized OHCA patients.

Variables	Total (n = 11000)	Non-discharge (n = 8501)	Discharge (n = 2499)	*P* value
Age (mean, SD)	70.6±15.7	71.7±15.2	66.7±16.7	<0.001
Male (n, %)	6424(58.4)	4949(58.2)	1475(59.0)	0.486
Liver failure (n, %)	209(1.9)	165(1.9)	44(1.8)	0.619
Heart failure (n, %)	3289(30.0)	2602(30.6)	687(27.5)	0.003
Renal failure (n, %)	2642(24.0)	2124(25.0)	518(20.7)	<0.001
Respiratory failure (n, %)	9590(87.2)	7610(89.5)	1980(79.2)	<0.001
Neurological failure (n, %)	2071(18.8)	1458(17.2)	613(24.5)	<0.001
Metabolic problem (n, %)	372(3.4)	312(3.7)	60(2.4)	0.003
Diabetes mellitus (n, %)	2615(23.8)	2034(23.9)	581(23.2)	0.501
Hypertension (n, %)	2184(19.9)	1620(19.1)	564(22.6)	<0.001
Stroke (n, %)	856(7.8)	676(8.0)	180(7.2)	0.235
CAD (n, %)	2042(18.6)	1361(16.0)	681(27.3)	<0.001
Valvular heart disease (n, %)	255(2.3)	157(1.8)	98(3.9)	<0.001
Cardiomyopathy (n, %)	184(1.7)	93(1.1)	91(3.6)	<0.001
Atrial flutter/fibrillation (n, %)	390(3.5)	237(2.8)	153(6.1)	<0.001
Pulmonary embolism (n, %)	42(0.4)	38(0.4)	4(0.2)	0.063
COPD (n, %)	742(6.7)	571(6.7)	171(6.8)	0.861
Asthma (n, %)	147(1.3)	110(1.3)	37(1.5)	0.538
Malignancy (n, %)	808(7.3)	692(58.4)	116(4.6)	<0.001

OHCA: out-of-hospital cardiac arrest; SD: standard deviation; CAD: coronary artery disease; COPD: chronic obstructive pulmonary disease.

Survivors were more likely to have been admitted to a higher hospital level with a higher volume. The geographic area of the admission hospital was associated with the rate of survival to discharge. In the discharge group, 89.7% of the hospitals were teaching hospitals, while 84.8% were teaching hospitals in the non-discharge group (*P*<0.001). Details of the hospital characteristics are given in Table 2.

**Table 2:** Hospital characteristics of hospitalized OHCA patients.

Variables	Total (n = 11000)	Non-discharge (n = 8501)	Discharge (n = 2499)	*P* value
Level of hospital (n, %)				<0.001
Medical center	2255(20.5)	1503(17.7)	752(30.1)	
Regional	6392(58.1)	5077(59.7)	1315(52.6)	
Local	2353(21.4)	1921(22.6)	432(17.3)	
Geographic area (n, %)				<0.001
Taipei city	1861(16.9)	1319(15.5)	542(21.7)	
Northern	3789(34.4)	2979(35.0)	810(32.4)	
Central	2080(18.9)	1578(18.6)	502(20.1)	
Southern	2822(25.7)	2281(26.8)	541(21.6)	
Eastern	382(3.5)	292(3.4)	90(3.6)	
Offshore	66(0.6)	52(0.6)	14(0.6)	
Teaching hospital status (n, %)	9450(85.9)	7208(84.8)	2242(89.7)	<0.001
Bed size (n, %)				<0.001
1^st^ quartile	2752(25.0)	2285(26.9)	467(18.7)	
2^nd^ quartile	2785(25.3)	2185(25.7)	600(24.0)	
3^rd^ quartile	2716(24.7)	2071(24.4)	645(25.8)	
4^th^ quartile	2747(25.0)	1960(23.1)	787(31.5)	
ICU bed size (n, %)				<0.001
1^st^ quartile	2793(25.4)	2349(27.6)	444(17.8)	
2^nd^ quartile	2747(25.0)	2166(25.5)	581(23.2)	
3^rd^ quartile	2719(24.7)	2047(24.1)	672(26.9)	
4^th^ quartile	2741(24.9)	1939(22.8)	802(32.1)	

OHCA: out-of-hospital cardiac arrest; ICU: intensive care unit.

As shown in Table 3, the percentage of patients diagnosed with VF on admission was 26.5% in the discharge group. In all, 7.4% subjects were transferred to another hospital; the percentage was higher in the discharge group (13.9%) than in the non-discharge group (5.5%) (*P*<0.001). Length of hospital stay was positively associated with prognosis. There was no significant difference in the distribution of patients who survived to discharge from 2007 to 2010 and from 2011 to 2013.

**Table 3:** Event characteristics of hospitalized OHCA patients.

Variables	Total (n = 11000)	Non-discharge (n = 8501)	Discharge (n = 2499)	*P* value
Cardiac rhythm on admission (n, %)				<0.001
Non-VF	9762(88.7)	7925(93.2)	1837(73.5)	
VF	1238(11.3)	576(6.8)	662(26.5)	
Transfer to another hospital (n, %)	817(7.4)	469(5.5)	348(13.9)	<0.001
Length of hospital stay (mean, SD)	13.3±30.5	10.2±28.9	23.6±33.2	<0.001
Calendar year (n, %)				0.221
2007 to 2010	5505(50.0)	4227(49.7)	1278(51.1)	
2011 to 2013	5495(50.0)	4274(50.3)	1221(48.9)	

OHCA: out-of-hospital cardiac arrest; VF: ventricular fibrillation; SD: standard deviation.

**Fig 1:**
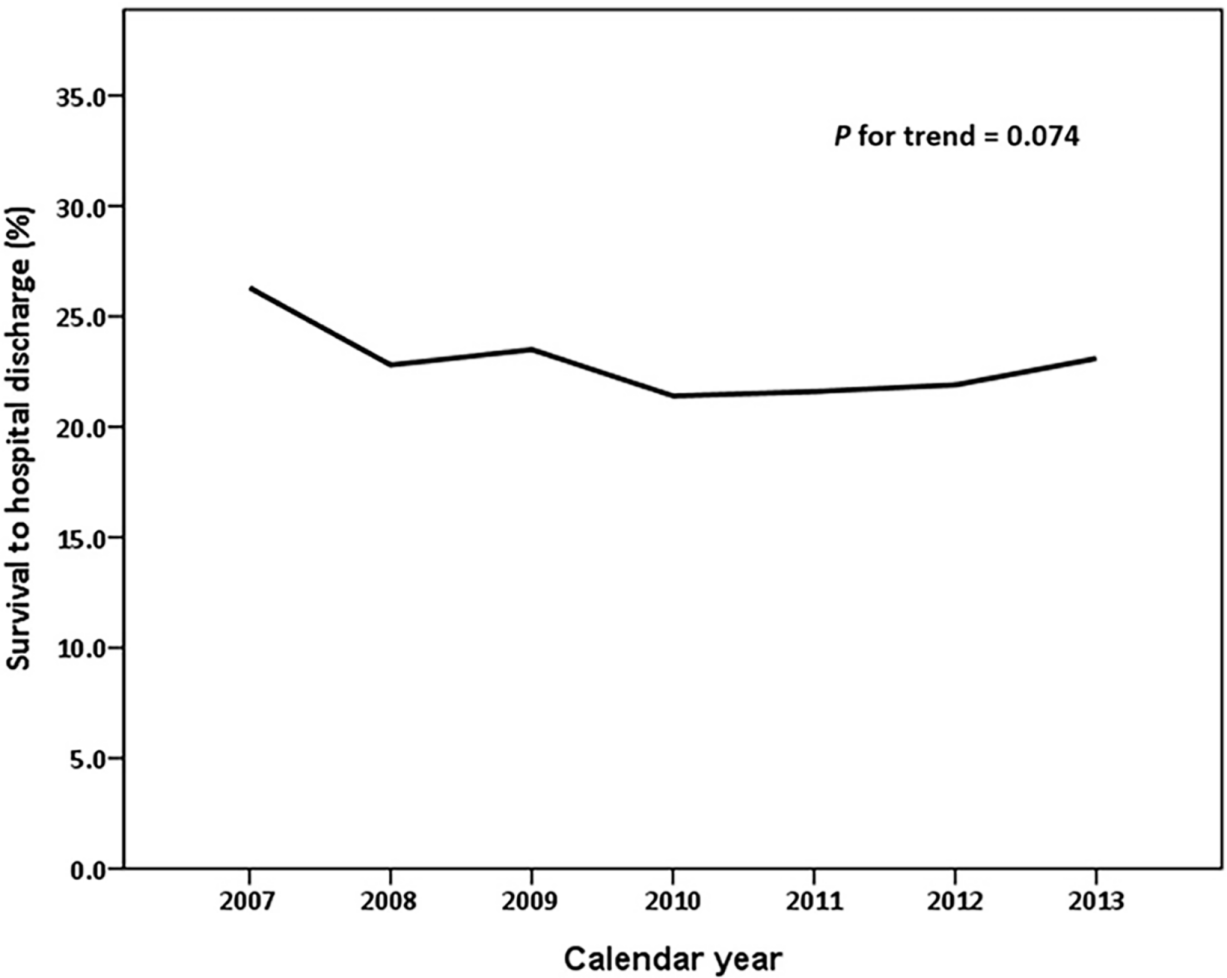
Temporal trend of survival to hospital discharge among hospitalized out-of-hospital cardiac arrest patients.

As shown in Table 4, multivariate analysis demonstrated that each yearly increment in age decreased the probability of survival to hospital discharge by 2% [adjusted odds ratio (aOR): 0.98; 95% confidence interval (CI): 0.98–0.99]. The number of failing organs was negatively related to survival to hospital discharge (aOR: 0.75; 95% CI: 0.71–0.80). Cardiac comorbidities were independent predictors of improved survival. In terms of the hospital level, patients admitted to a medical center had a better prognosis than those admitted to a regional hospital (aOR: 0.76; 95% CI: 0.67–0.87). Cardiac rhythm was a strong predictive factor associated with survival to discharge (VF vs. non-VF: aOR: 3.51; 95% CI: 3.06–4.01). Patients transferred to another hospital were 59% more likely to survive to discharge (aOR: 1.59; 95% CI: 1.30–1.93). Meanwhile, the aOR of survival to discharge was 1.01 for each daily increment in hospital stay (aOR: 1.01; 95% CI: 1.01–1.02). The rate of survival to hospital discharge was not significantly elevated by calendar year (*P* for trend = 0.074) (Fig 1).

**Table 4:** Survival factors for hospitalized OHCA patients.

Variables	Group comparison	aOR^a^	95% CI	*P* value
Age		0.98	0.98–0.99	<0.001
Number of failing organs		0.75	0.71–0.80	<0.001
CAD	Yes vs. no	1.49	1.33–1.68	<0.001
Valvular heart disease	Yes vs. no	1.48	1.12–1.97	0.006
Cardiomyopathy	Yes vs. no	1.55	1.12–2.14	0.008
Atrial flutter/fibrillation	Yes vs. no	1.50	1.18–1.89	0.001
Malignancy	Yes vs. no	0.58	0.47–0.72	<0.001
Level of hospital	Regional vs. medical center	0.76	0.67–0.87	<0.001
	Local vs. medical center	0.88	0.73–1.07	0.222
ICU bed size	2^nd^ vs. 1^st^ quartile	1.33	1.15–1.54	<0.001
	3^rd^ vs. 1^st^ quartile	1.59	1.35–1.87	<0.001
	4^th^ vs. 1^st^ quartile	1.56	1.31–1.86	<0.001
Cardiac rhythm on admission	VF vs. non-VF	3.51	3.06–4.01	<0.001
Transfer to another hospital	Yes vs. no	1.59	1.30–1.93	<0.001
Length of hospital stay		1.01	1.01–1.02	<0.001

^a^Variables selected for the model: age, number of failing organs, hypertension, CAD, valvular heart disease, cardiomyopathy, atrial flutter/fibrillation, malignancy, hospital level, geographic area, teaching hospital status, bed size, ICU bed size, cardiac rhythm on admission, transfer to another hospital, length of hospital stay.

OHCA: out-of-hospital cardiac arrest; aOR: adjusted odds ratio; CI: confidence interval; CAD: coronary artery disease; ICU: intensive care unit; VF: ventricular fibrillation.

## Discussion

In our study, 2,499 subjects survived to hospital discharge, with an overall rate of 22.7%, from 2007 to 2013 in Taiwan; however, the temporal trend of survival to hospital discharge was static over the study period. Cardiac rhythm coded VF on admission was a confirmed dominant independent variable.

Previous results have shown survival to vary widely from 8.3% to 38.0% [23, 26, 27]. Although we extracted subjects using ICD-9-CM codes, the inclusion/exclusion criteria made little difference due to the database characteristics. In addition, studies have recruited different types, such as those resuscitated by EMS, treated by teams in the hospital ED, or surviving to hospital admission. To elucidate the effect of post-cardiac arrest care, we only analyzed the survival of OHCA patients admitted to a hospital; the do-not-resuscitate (DNR) policy is not widely selected in Taiwan, which could result in discrepancies with the results of other studies. However, aside from this issue, post-cardiac arrest care indirectly influences survival in various ways.

Previous results have indicated that the rate of OHCA survival has not dramatically changed for decades [12]. In our study, the temporal change in the rate of survival to hospital discharge showed no significant improvement, similar the results of a study performed in Canada [27]. Nevertheless, data from Sweden indicated that out-of-hospital death decreased by 4% each year [28], and the in-hospital mortality rate of patients admitted to a hospital for cardiac arrest decreased by 11.8% in the United State [25]. After implementation of the chain of survival concept for post-resuscitation care, one-month survival with a favourable neurological outcome in OHCA patients increased by 7.8-fold [6]. There are potential explanations for the discrepancies in these temporal trends. First, most studies have focused on the survival improvement of OHCA patients suffering from conditions with a cardiac aetiology, and it has been shown that a condition having a cardiac origin was a positive predictor for outcome. Second, eligible patients were more likely to include survivors transported from an out-of-hospital location to an ED. In our study and in a former study [27], only subjects who survived to hospital admission were tracked. Finally, such non-identical outcomes seemed to have led to different conclusions. In our study, the rate of survival to hospital discharge was applied to demonstrate the effectiveness of post-cardiac arrest care.

Recognizing different factors may help to improve post-cardiac arrest care. As found by previous studies, age was a predictor of outcome [25, 29]. We found that the mean age of the discharge group was 5 years younger than that of the non-discharge group. Our results were also consistent with those of several studies in that the risk of in-hospital mortality increased with the number of failing organs and comorbidities [23, 25]. However, the incidence of neurological failure was higher in the discharge group. This result is probably because patients who survived to discharge tended to have symptoms of neurological impairment recorded during their hospitalization. In other words, non-survivors might expire before being evaluated for neurological function. Consistently, the results of a previous study indicated a higher percentage of survival to discharge among OHCA patients with mild/moderate neurological and performance disability [16]. Because there were no data regarding cerebral performance category score [30], we could only track neurological failure by ICD-9-CM code, which does not indicate the severity of brain damage. Cardiac comorbidities were likely associated with VF or ventricular tachycardia (VT); thus, they were rationally positive predictors of survival [31]. Our findings were similar to those of Dudas et al., who reported that malignancy was a risk factor for survival [28]. Since 2000, when the Hospice Palliative Care Act was passed in Taiwan, the DNR policy has prevented unnecessary CPR. Therefore, the CPR success rate was lower in cancer patients than in non-cancer patients in Taiwan, and cancer seemingly had a negative effect on survival [32].

In larger hospitals, patients benefit from higher intensity service, more rapid action, and new emerging technology. The present study found that a higher volume of ICU beds was strongly associated with survival to hospital discharge, while other hospital characteristics have been linked to in-hospital mortality and discharge among OHCA patients [5, 27, 33, 34]. Therefore, variations in the delivery and quality of healthcare usually result in different OHCA survival outcomes. In accordance with previous observations, our analysis revealed that the rate of discharge increased with ICU bed number. However, the variables hospital level, bed number, and ICU bed number might have redundant effects on the primary outcome. Our analysis showed that ICU bed number is likely to be a much better independent predictor in post-cardiac arrest care.

International findings revealed that the initial cardiac rhythm of patients, VF/VT, has an enormous impact on OHCA survival [7, 29]. In this context, cardiac rhythm on admission was also a profound factor correlated with survival to discharge. Hospitalized patients with VF were more than three times as likely to survive to discharge than those with a non-VF rhythm. In our study, 11.3% of OHCA patients who survived to hospital admission presented with VF. However, studies have illustrated higher percentages of VF/VT. Data from New Zealand showed that close to half of cardiac arrest patients presented with VF [15]. Approximately one quarter of OHCA patients in Sweden presented with a VF/VT rhythm [35, 36]. These differences might be because we retrieved cases using the primary diagnostic code assigned upon hospital admission, and information regarding the initial cardiac rhythm occurring before admission might be lost. Furthermore, in OHCA, a secondary rhythm is likely to be present on admission. Additionally, the studies mentioned above limited their subjects to those with OHCA of a cardiac origin and a higher proportion of VF/VT rhythms. In summary, our finding that cardiac rhythm on admission influenced survival is in agreement with previous reports. Two other variables, transfer to another hospital and length of hospital stay, were positively associated with survival. In clinical practice, resuscitated OHCA patients with the return of spontaneous circulation are sent to the ICU for advanced nursing care. If patients are diagnosed with this event in a small hospital with little infrastructure, they would be transferred to a well-equipped and experienced care center or a larger hospital. In addition, stable subjects who survived were more likely to have been transferred to another hospital closer to their home than were non-survivors in Taiwan. Thus, survivors, who recovered better, tended to stay longer in a hospital than non-survivors [23,24,34], which might be why referral to another hospital and the length of hospital stay contributed to a positive outcome. In the future, more studies of patient movement between hospitals should investigate this issue further.

### Limitations

There are some limitations and weaknesses in our study. First, the outcome of interest was clearly defined as survival to hospital discharge. Even if we calculated the rate of neurological failure among the comorbidities, data regarding survival with functional recovery among hospitalized OHCA patients were not available. The collection of unfavorable neurological data could neutralize the effectiveness of post-cardiac arrest care. Second, the current study was hindered by an inability to follow survival outcomes after discharge. Therefore, we could not examine the long-term survival of patients [37]. Next, clinical characteristics were obtained from secondary diagnostic codes and operational procedure codes, which lack detailed information. Thus, we could not distinguish whether certain diagnoses were causes, pre-existing conditions or comorbidities, which precludes the establishment of a causal relationship between clinical characteristics and outcomes. Third, rapid transport to a hospital for advanced life support is one determinant of survival. The policy for treating OHCA in Taiwan is rapid transport to a nearby hospital, regardless of hospital level, volume, or teaching status. Due to dataset restrictions, no information about travel time to treatment was available in this study. If patients are delayed in reaching a hospital, there is an overwhelmingly high probability of them dying at the scene or in the ED, and these patients could not be included in the analytical process. Therefore, travel time to treatment is less likely to affect the conclusion. Finally, our analysis using cardiac rhythm on admission does not account for patients whose rhythm occurred at the onset of cardiac arrest. It is likely that we underestimated the proportion of VF/VT among cardiac rhythms, thereby weakening its effect on survival to discharge.

## Conclusions

In conclusion, we utilized a structural framework to investigate OHCA survival. No differences in survival were found between the examined calendar year periods. The results show that factors involved in cardiac comorbidities, hospital level, ICU bed number, cardiac rhythm on admission, transfer to another hospital and length of hospital stay have a significant positive association with survival to hospital discharge. Our findings also suggest that age, number of failing organs, and malignancy are negatively related to survival to discharge.
